# Duodenal descending part-jejunum intussusception and upper gastrointestinal bleeding caused by duodenal fibrolipoma: a case report

**DOI:** 10.1186/s12893-019-0634-1

**Published:** 2019-11-12

**Authors:** Bangbo Zhao, Xingtong Zhou, Weibin Wang

**Affiliations:** 1 Department of General Surgery, Peking Union Medical College Hospital, Chinese Academy of Medical Science, Beijing, China; 2 Department of Surgery, Peking Union Medical College Hospital, Chinese Academy of Medical Science, Beijing, China

**Keywords:** Duodenal fibrolipoma, Duodenum-jejunum intussusception, Upper gastrointestinal bleeding

## Abstract

**Background:**

Duodenal fibrolipoma and duodenum-jejunum intussusception are both rare occasions in clinical practice. The diagnosis of duodenal fibrolipoma mainly depends on endoscopy examination, supplemented by CT and MRI. As the tumor grows, some severe symptoms need surgical intervention. As the development of endoscopic techniques, the operation plan should be made individually.

**Case presentation:**

A 47-year-old female with the complaint of upper abdominal pain and melena was reported. Abdominal examination revealed upper abdomen lightly tender and blood test showed severe anemia. Image and endoscopy examination exhibited “a giant mass” in the descending (D2) part of duodenum, dragged by the tumor into the distal intestinal canal and causing intussusception. Intermittent blood transfusion treatment, enteral and parenteral nutrition were adopted to adjust her general state. Two weeks later, the mass was resected together with the basement intestinal wall via the jejunum incision and then the intussuscepted D2 part was restored. The paraffin pathological diagnosis correlated with the preoperative judgment of fibrolipoma and the patient was discharged healthy on POD 14.

**Conclusions:**

Duodenal fibrolipoma is a rare disease, infrequently causing intussusception and severe upper GIB. Duodenoscopy and endoscopic ultrasound contribute to making an appropriate diagnosis, and for patients with severe symptoms needed surgical intervention, operation plan should be individualized depending on the size and location of the lesion.

## Background

Gastrointestinal fibrolipoma, especially occurring in duodenum, is a kind of benign tumor of extremely low morbidity and detection rate, accounting for only 5–6% of all gastrointestinal tumors [1]. Giant duodenal fibrolipoma may cause upper abdominal discomfort, GIB, intussusception and even intestinal obstruction. Duodenum-jejunum intussusception is extremely rare as well on account of the short mesentery of duodenum as a retroperitoneal organ. Here, we present a rare giant duodenal fibrolipoma causing D2 part-jejunum intussusception and upper GIB.

## Case presentation

The patient, a 47-year-old female, was admitted to our hospital with the complaint of upper abdominal pain and melena for 1 month. After once eating oysters 1 month ago, the patient developed a cold feeling with pain in the upper abdomen. One week later, she gradually developed acid reflux, melena and reduced stool volume, whose frequency of melena was 2–3 times a day. In terms of physical examination, the whole abdomen was soft, no gastrointestinal type and peristaltic wave were seen, the upper abdomen was lightly tender, no mass was touched, and the bowel sounded normal. Admission test showed severe anemia (HGB 47 g/L), with liver and kidney function and tumor markers normal. Intermittent blood transfusion treatment, enteral and parenteral nutrition were adopted along with the appearance of nearly complete intestinal obstruction.

**Fig. 1:**
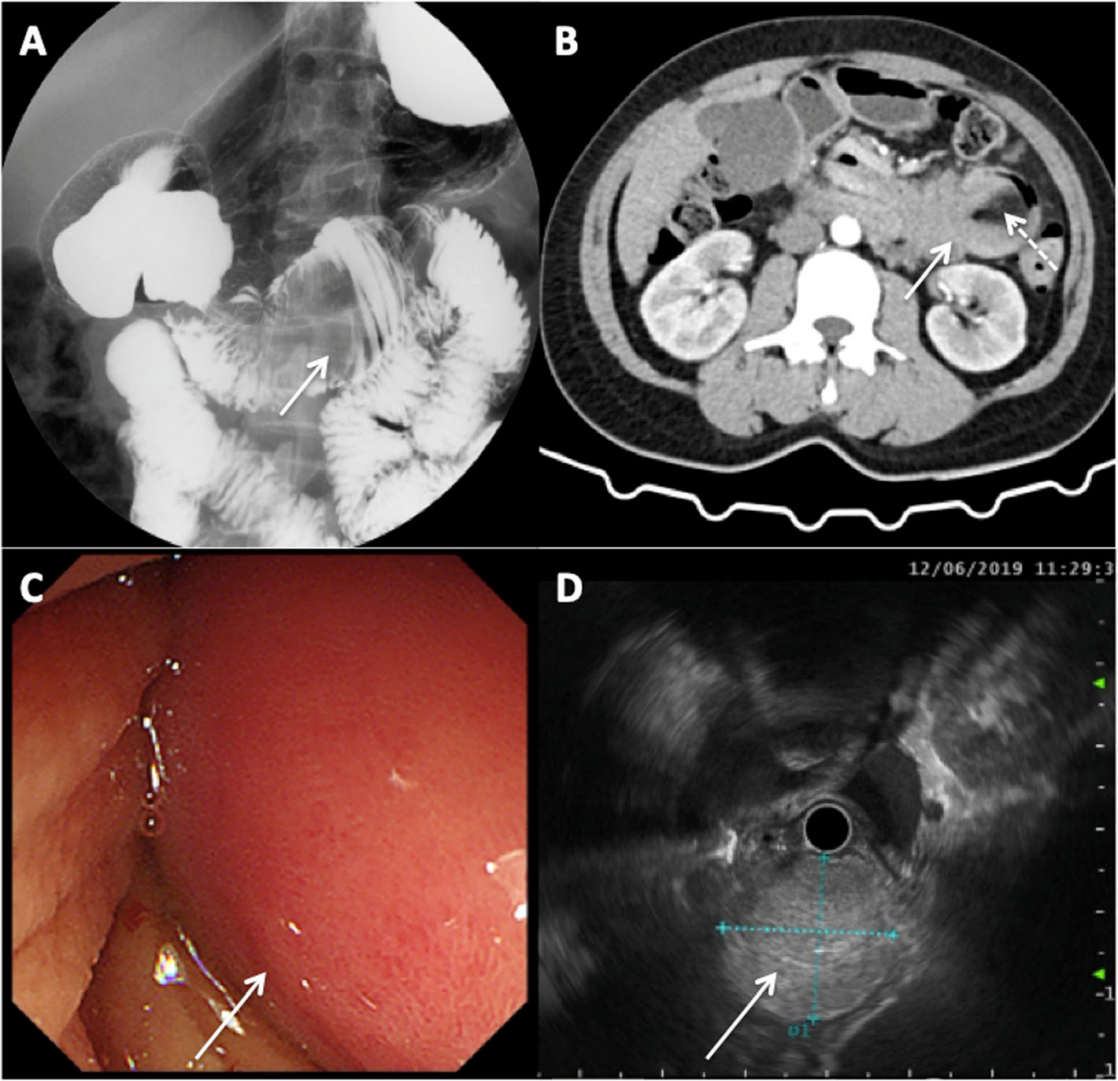
Endoscopy and radiography examination. **a** Upper gastrointestinal tract showed filling defect (arrow) in the horizontal and ascending part of duodenum; **b** Abdominal enhanced CT showed a cuff-like change (solid arrow) of intestinal segments in the left upper abdomen with fat-density opacity (dashed arrow) seen in the lumen; **c** Duodenoscopy showed “a large mass” (arrow) in descending part; **d** Ultrasound endoscopy showed a medium-high echo mass (arrow) derived from the submucosa)

The upper digestive tract radiography showed a mass-like filling defect in the D3 and D4 part of duodenum, and no obvious damage of mucosa was observed, considering the possibility of a submucosal lesion (Fig. 1a). Abdominal enhanced CT showed a cuff-like change of intestinal segments in the left upper abdomen, and a spindle-shaped fat-density opacity (3.6 × 3.7 × 6.7 cm) was seen in the lumen (Fig. 1b), whose boundary was clear and no enhancement was observed in the enhanced scan. The D2 part and its proximal side were pulled, which correlated the performance of the fibrolipoma combined with intussusception. The patient underwent duodenal endoscopy further, which showed stenosis of the access into the D2 part, where “a large mass”, soft and with smooth surface mucosa, was seen in the lumen (Fig. 1c). Nevertheless, there was no active bleeding seen in view. Ultrasound endoscopy showed echo of the mass was medium to high, with the internal echo uniform (Fig. 1d). Moreover, the lesion seemed to continue with the submucosal layer of the intestinal wall, and did not involve the junction of the pancreaticobiliary duct. In order to further confirm bleeding site, ^99^mTc-RBC gastrointestinal bleeding site determination was performed, which showed the 6th group of intestine would be the bleeding site and no abnormal radioactive elevation was observed in duodenum and proximal jejunum.

**Fig. 2:**
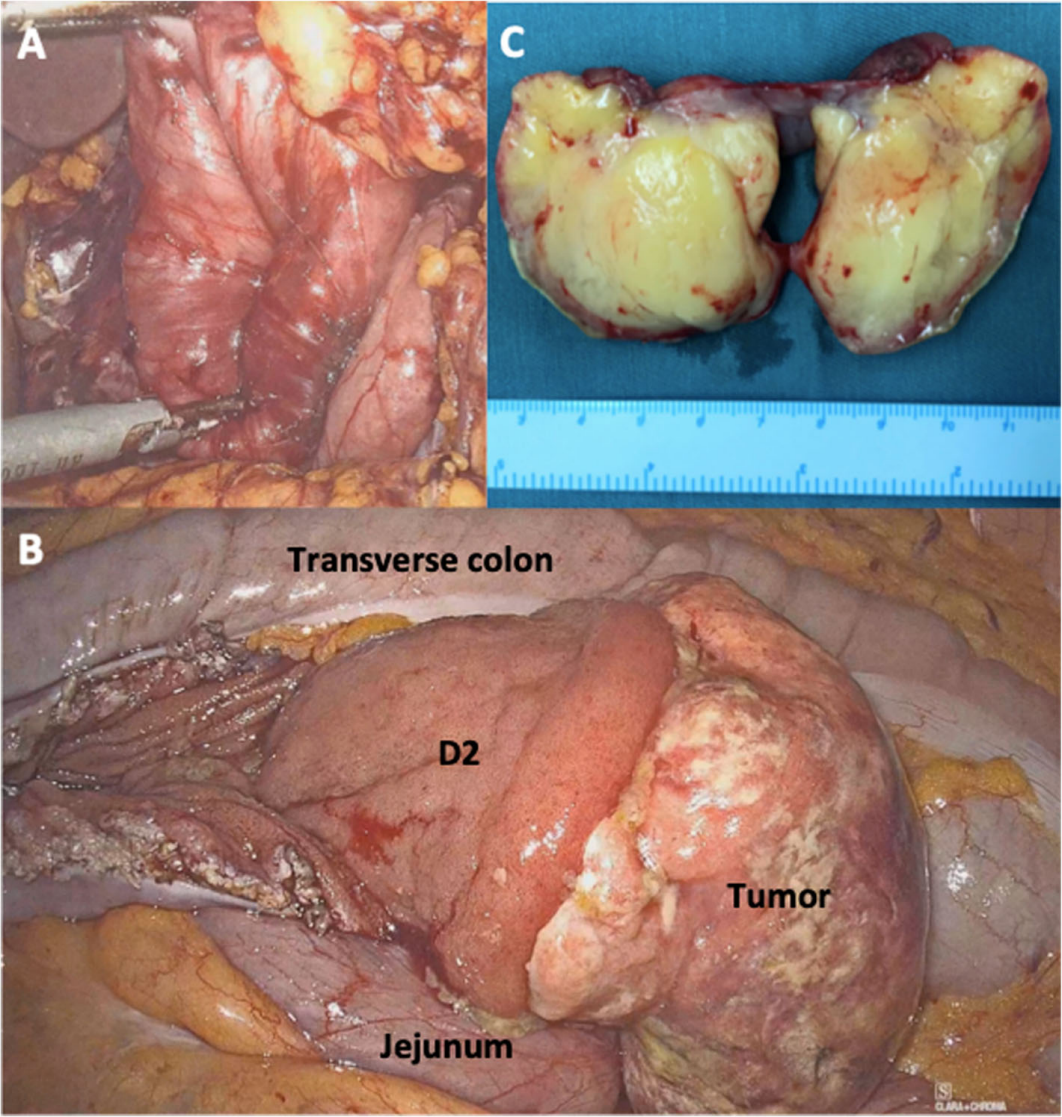
Operation procedure and the specimen. **a** Vortex sign in the primary site of tumor (the descending part of duodenum); **b** Duodenal descending part mass revealed after cutting jejunum; **c** Profile view of the mass

An Elective laparoscopic exploration was performed. Intraoperatively, a mass, about 5 × 4 cm in diameter, was found in the jejunum near Treitz ligament, and after opening the duodenal collateral ligament, the D2 part of duodenum was found incarcerated into the D3 part, D4 part and jejunum, which presented the “vortex sign” (Fig. 2a). Cutting the jejunum open, the pedicle of this mass was wide, the surface was brittle, and it was easy to bleed when touching (Fig. 2b). For prudential reasons, intestine till ileocecal junction was explored, segmental bloody content was nearly equally distributed, which hint the bleeding site was the mass. And therefore, a median small incision was made into the abdomen to completely remove the mass together with the basement intestinal wall via the jejunum incision (Fig. 2c). The frozen pathological diagnosis was adipose-derived tumor without obvious cell atypia. The intussuscepted D2 part with abundant blood supply was then restored, and the two incisions in the intestine were closed appropriately, with the lumen unobstructed and without blood supply disorder. The paraffin pathological diagnosis of this lesion was fibrolipoma, and the result of immunohistochemical staining was AE1/AE3 (partially +), Ki-67 (index 10%), SMA (+), S-100 (+) and CD34 (vascular +). The patient was removed from the duodenal drainage on POD 2 and oral feeding was started on POD 7. After successful transition to a semi-liquid diet on POD 10, the patient was discharged from the hospital on POD 14. The HGB was gradually increased and stabilized before discharge at around 100 g/L, and there were no significant complications occurring such as infection, hemorrhage and intestinal fistula perioperatively.

## Discussion and conclusions

Duodenal fibrolipoma is a rare benign tumor of the small intestine, accounting for 28.6% of small intestinal fibrolipoma [2]. 80% of duodenal fibrolipoma occurred in age of 40–70 without significant gender difference, and predilection part in duodenum of the disease was the D2 part (39%) [3]. On account of the difficulty in early detection, this kind of masses often develop into large bulging lesions. As a result of food friction and gastric acid erosion, congestion, erosion and ulceration of the lesion surface cause the main clinical manifestations of this disease, long-term repeated gastrointestinal bleeding, upper abdomen discomfort, acid reflux and hiccups. Scarcely, the junction of the pancreaticobiliary duct involved may cause obstructive jaundice or acute pancreatitis, and the asymmetric peristalsis caused by fibrolipoma may also trigger rare stomach-duodenum or duodenum-jejunum intussusception [4].

The combination of endoscopic orthoptic findings and endoscopic ultrasonography findings, the clinical diagnosis of duodenal fibrolipoma can be basically confirmed [5]. Because the distal part (D3, D4 part) of duodenum is not the routine examination scope of gastroscopy, it is easily to be miss-diagnosed. Endoscopic ultrasound is of the most diagnostic value for duodenal fibrolipoma. Dense hyperechoic lesions originating from submucosal, with clear borders and uniform internal echo can distinguish this disease from the majority of other duodenal diseases. The biopsy pathology of duodenal fibrolipoma is mostly chronic mucosal inflammation, indicating that endoscopic biopsy may be not useful for diagnosis [3]. CT and MRI can assist in identifing the fat components inside the lesion and recognizing the relationship between the lesion and the surrounding tissue. Surgical treatment may be considered for patients with unclear diagnosis or severe symptoms. The choice of operation plan depends mainly on the size of the lesion and its relationship with duodenal papilla. Small lesions are feasible for endoscopic snare resection [6] and laparoscopic or open tumor resection or partial bowel resection are optional for large lesions without involvement of pancreaticobiliary junction [7]. There are reports of endoscopic submucosal resection for large lesions as well [5, 8]. As for lesions involving pancreaticobiliary duct, pancreaticobiliary angioplasty ought to be performed while resecting the tumor, or in full weighting, adopt the pancreaticoduodenectomy [9].

In this case, the duodenal fibrolipoma was characterized that the lesion originating from the D2 part gradually drove the local intestine sheath into the D3 part at the beginning of occurrence. As the lesion grew, the tumor was “incarcerated”, which made the D2 part difficult to be restored and formed duodenum-jejunum intussusception finallly. Because the duodenum is a retroperitoneal organ and the duodenal mesentery is short, its position is relatively fixed and less intussusception occurs. Once it is intussuscepted, it usually combines with a large mass inside or congenital small intestine dysplasia. Duodenum-jejunum intussusception previously reported often involved the D3 and D4 part of duodenum and our case shoule be the first report of D2 part-jejunum intussusception [9].

Moreover, the patient was combined with severe anemia which was difficult to be corrected by blood transfusion. As a matter of fact, our preoperative examination, duodenal endoscopy and ^99^mTc-RBC gastrointestinal bleeding site determination, both failed in detecting the real bleeding site. For endoscopy, its body was blocked by the tumor in the D2 and D3 part and the “mass” we saw was in fact the adjacent mucosa. Then for nuclear scan, as the tumor bled intermittently, the residual blood affected the accuracy of the result.

In summary, duodenal fibrolipoma is a rare disease, which should be considered when encountered with the unexplained upper gastrointestinal bleedind. Duodenoscopy and endoscopic ultrasound should be conducted to confirm the diagnosis, and for patients with severe symptoms, surgeons should take surgical intervention and individualize the surgical plan.

## Data Availability

All data are included in the section of Case Presentation and are available from the corresponding author on reasonable request.

## Abbreviations

CT: Computer tomography
D2 part: duodenal descending part
D3 part: duodenal horizontal part
D4 part: duodenal ascending part
GIB: Gastrointestinal bleeding
HGB: Hemoglobin
MRI: Magnetic resonance imaging
POD: Postoperative day
RBC: Red blood cell

## References

[1] Wichendu PN, Dodiyi-Manuel A (2013). Gastric outlet obstruction from duodenal lipoma in an adult. Niger J Surg.

[2] Zhu R, Luo YF, Wu HW (2018). Small intestinal lipoma: a clinicopathologic analysis of 14 cases. J Diag Pathol.

[3] Pei MW, MR HU, Chen WB (2017). Diagnosis and treatment of duodenal lipoma: a systematic review and a case report. J Clin Diagn Res.

[4] Chen HT, Xu GQ, Wang LJ (2010). The diagnosis and treatment of duodenal lipoma. Chin J Intern Med.

[5] Xu MD, Li L, Wang XY (2012). Endoscopic submucosal dissection in the treatment of giant gastrointestinal lipomas. Chin J Dig.

[6] Thorlacius Henrik, Weiber Håkan, Ljungberg Otto, Nielsen Jörgen, Toth Ervin (2013). Endoscopic diagnosis and treatment of a giant duodenal lipoma presenting with gastrointestinal bleeding. Endoscopy.

[7] Parmar AK, Bibyan M, Khandelwal R (2013). Laparoscopic management of a large duodenal lipoma presented as gastric outlet obstruction. JSLS.

[8] Aslan F, Akpinar Z, Cekic C (2015). En bloc resection of a 9 cm giant gastro-duodenal lipoma by endoscopic submucosal dissection. Dig Liver Dis.

[9] Chai LF, Batista PM, Lavu H (2016). Taking the Lead: a case report of a leiomyoma causing Duodeno-duodenal intussusception and review of literature. Case Rep Pancreat Cancer.

